# Pathophysiology of Concussive Non-Penetrative Captive Bolt Stunning of Turkeys

**DOI:** 10.3390/ani9121049

**Published:** 2019-11-29

**Authors:** Troy J. Gibson, Emma King, Jade Spence, Georgina Limon

**Affiliations:** 1 Department of Production and Population Health, Royal Veterinary College, Hatfield AL9 7TA, UK; emma.king@poultryhealthservices.com (E.K.); or georgina.limon-vega@pirbright.ac.uk (G.L.); 2 Humane Slaughter Association, Wheathampstead AL4 8AN, UK; jade@hsa.org.uk

**Keywords:** non-penetrating captive bolt, stunning, turkey, behaviour, pathology

## Abstract

**Simple Summary:**

For routine slaughter, animals are rendered unconscious prior to the act of slaughter in order to prevent pain and distress. The electrical stunning methods generally used by small-scale seasonal turkey producers can be ineffective in inducing unconsciousness. An alternative method is non-penetrating captive bolt stunning (NPCB) which involves the firing of a mushroom shaped metal rod against the head to induce concussion and unconsciousness. This study evaluated the effectiveness of two models of NPCB guns for inducing unconsciousness in turkeys. Effectiveness was assessed with behavioural responses and the level of induced brain damage. Both NPCB guns used in this study were effective in inducing unconsciousness as assessed with behavioural responses. There was extensive damage throughout all regions of the brain. These results support the use of NPCB stunning as an effective stunning method for commercial slaughter of turkeys when used correctly.

**Abstract:**

The non-penetrative captive bolt (NPCB) has been proposed as a more humane and practical alternative to constant voltage electrical stunning for small-scale seasonal turkey producers. This study evaluated the effectiveness of the CASH^®^ Small Animal Tool (SAT) (formerly known as the CASH^®^ Poultry Killer, CPK) and three configurations of the Turkey Euthanasia Device (TED), assessing behavioural, cranial/spinal responses and brain pathology. Immediately after stunning all birds showed cessation of rhythmic breathing and loss of neck and beak tension. One bird shot with the TED/hen configuration showed a positive nictitating membrane reflex in one eye with no other positive reflexes. All birds had moderate/severe gross damage to the hyperpallium layer over the cerebrums. For almost all other cerebrum structures, thalamus, and hindbrain, the TED/poult configuration and SAT produced the most extensive damage. The frequency of petechial haemorrhage in the pons and medulla was less in SAT shot birds (76% and 71% respectively) compared to those shot with the different configurations of the TED (ranging from 81% to 100%), however this difference was not significant. In conclusion, both NPCB guns were effective in inducing unconsciousness in turkeys, regardless of the variations in shot position and the different configurations of the TED.

## 1. Introduction

Prior to slaughter for human consumption, turkeys are stunned with variations of three main methods. These are electrical stunning (waterbath head-to-shackle, head-only, and non-waterbath head-to-body), controlled atmospheric stunning (CAS; CO_2_ in different concentration phases and/or with mixtures of inert gases) and captive bolt (penetrative and non-penetrative). The aim is to render the animal unconscious and insensible to pain prior to, during and after the severance of neck tissues (including bilateral severance of the carotid arteries and jugular veins). For small-scale seasonal producers CAS systems are not currently feasible due to their high capital and running costs, waterbath systems can also be prohibitive due to cost and issues associated with their effectiveness. As an alternative to waterbath stunning a large proportion of seasonally produced birds are stunned with constant voltage head-only systems. However, these systems are reversible, meaning if a secondary (killing) procedure is not performed very soon after stunning the bird will recover consciousness. Furthermore, research in chickens (*Gallus g. domesticus*) has reported that the duration of induced unconsciousness varied between birds, with some recovering within 9 s (range 9 to 31). This short period of recovery could result in some birds regaining consciousness during or prior to exsanguination [1].

An alternative stunning method is the non-penetrative captive bolt (NPCB). These are generally powered by compressed air, gun powder, or propane. With sufficient airline pressures or powerloads and when placed in the correct position (top of the head on the midline between the eyes and ears), they can be effective in inducing unconsciousness in turkeys and chickens without recovery [2,3,4,5], while variation from the ideal shooting position and inconsistencies in powerloads was reported to result in incomplete concussion in slaughter weight turkeys following stunning with three different NPCB guns, using electroencephalographic (EEG) and behavioural indices to evaluate unconsciousness [4]. Erasmus et al. [6] and Woolcott et al. [5] examined pathology only or pathology and behaviour respectively after NPCB stunning in turkeys and concluded that pneumatic NPCB stunning was effective in producing profound damage to the brain, which was hypothesised to be responsible for death. Despite the above research, further work is needed to evaluate NPCB in commercial conditions as an appropriate and humane stunning method for small scale turkey seasonal producers.

The aim of this study was to assess the behavioural and cranial/spinal responses of turkeys shot during commercial production with two NPCB models (gunpowder and propane powered) and examine the pathophysiology of the resulting brain damage at the gross and microscopic level.

## 2. Materials and Methods

The study received local ethical approval from the Royal Veterinary College Ethics and Welfare Committee (URN 2019 1934-2R). Seventy-two turkeys (*Meleagris gallopavo*) from a seasonal producer in Southern England were stunned and slaughtered as part of routine commercial slaughter by two licensed slaughtermen using two NPCB gun models. These were the Accles & Shelvoke .22 CASH^®^ Small Animal Tool (SAT) (formerly known as the CASH^®^ Poultry Killer, CPK) (*n* = 27) and Bock Industries Turkey Euthanasia Device (TED) (*n* = 45).

The turkeys were a mix of Norfolk Bronze and Blacks (female 59, male 13) and were 23 weeks old. Turkeys were caught immediately prior to stunning and individually restrained in an inverted metal cone, with the back of the bird facing the slaughterman. Each bird’s head was suspended in air and held by the beak during shooting. The SAT was fired with the .22 brown 1.0 gr black-powder cartridges and the TED was powered by a propane fuel cell (Paslode, Illinois Tool Works Inc., Glenview, IL, USA), which was replaced at the start of each day regardless of the number of animals previously stunned with the fuel cell. Cartridges for the SAT were weighed on a precision balance (Acculab Vicon Balance, Sartorius AG, Göttingen, Germany) prior to and after shooting, only cartridges weighing above 0.723 g were included in the study.

**Figure 1:**
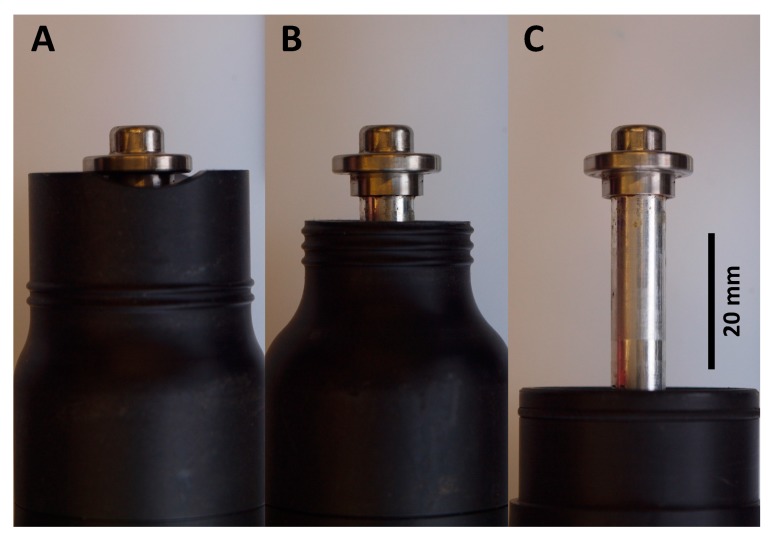
Total muzzle travel with the three configurations of the TED: (**A**) hen adaptor (9 mm); (**B**) modified poult adaptor (17 mm); and (**C**) no adaptor (45 mm).

When stunning, the muzzle of the captive bolt guns were placed on the surface of the top of the cranium at a perpendicular angle, with the head restrained by the free hand holding the beak. The muzzle was positioned on the midline of the head, between the eyes and the ears. The TED was used on 45 turkeys with three different configurations: (i) hen adaptor (9 mm muzzle travel) (*n* = 28); (ii) modified poult adaptor with 7 mm removed from the end to increase bolt travel (17 mm muzzle travel) (*n* = 11), the original unmodified poult adaptor was found to be ineffective during a pilot study; and (iii) the largest birds were shot with no adaptor (45 mm muzzle travel) (*n* = 6) (Figure 1). Meanwhile the SAT was used on 27 turkeys with the convex knocker head. Apart from the largest birds the treatment order and bird selection were randomised. For both the SAT and TED a decibel reading was recorded for each shot as an indirect measure of powerload performance. The decibel meter (Model CR303, Cirrus Research plc, UK) was placed 100 mm left of the captive bolt gun muzzle.

Immediately after shooting, all animals were assessed for clinical signs of sensibility, including the presence or absence of tonic and clonic convulsions, rhythmic breathing, nictitating membrane reflex, eyeball rotation, nystagmus, neck tension, and beak tension [1,4]. Eye-based reflexes were tested on both eyes. When the eyeball was covered in blood it was not possible to test for eye-based reflexes (one eye affected in 2 birds). Immediately after assessment of stun performance the animals were bled with bilateral severance of the carotid arteries and jugular veins. Time between stunning and exsanguination was recorded. Following death, birds were weighed and the heads removed and preserved in individually-labelled pots with 10% buffered formalin and left to fix for 5 months.

Following fixation, the skin over the head was removed and the location of the shot site measured in relation to bregma. The extent of skull fractures, subcutaneous haemorrhage and brain protrusion were assessed. Skull fractures were defined by the type of fracture and severity was subjectively assessed using a modified version of the scoring system used by Erasmus et al. [6]: None (no fractures intact skull), mild (1–2 hairline fractures, including partial depression), moderate (full depression and/or 3–5 complete fractures), and severe (full depression and >5 complete fractures, full fragmentation of the cranium). Protrusion of brain tissue out of the wound cavity was subjectively assessed as none, mild (≤1 mL of brain tissue), moderate (2–3 mL of brain tissue), or severe (>4 mL of brain tissue).

The skulls with the brain in-situ were sawn with an Oscillating Autopsy Saw with 64 mm Circular Autopsy Blade (Medezine Ltd., Sheffield, UK) into four sagittal cross-sections (approximately 3–4 mm thick), with the brain sections assessed and photographed in-situ prior to extraction of the tissue. Brains were examined both in-situ and after extraction for gross damage to specific neurological structures, displacement of tissues, and haemorrhage. Brain structures were classified according to the Avian Brain Nomenclature Forum system [7,8]. Data from the left and right hemispheres were pooled to aid analysis. Severity of tissue damage to specific brain regions (cerebrum, thalamus, hypothalamus, midbrain, pons, medulla, and cerebellum) was subjectively assessed as none (0%), mild (1%–20%), moderate (21%–49%) and severe (≥50%) damage [9,10,11].

Sagittal sections from the left and the right side of each brain were embedded in wax, stained with haematoxylin and eosin (H&E), sliced and placed on slides for histopathological assessment of damage, using light microscopy. Assessment of gross and histopathology was performed blinded to the treatments.

### Statistical Analysis

Descriptive statistics were conducted stratified by type of treatment, brain regions, and degree of brain damage. The extent to which birds’ weight was correlated with haemorrhagic damage and brain protrusion was assessed using analysis of variance (ANOVA). Decibel levels were non-normally distributed and were analysed with a Mann–Whitney test. For further statistical analysis, categorical variables representing the degree of brain damage and fracture classification were re-categorised (none and mild vs. moderate/severe). Similarly, treatment groups were collapsed to SAT and TED. Pearson’s Chi squared tests (or Fisher’s Exact tests where appropriate) were used to test for association between treatment group (SAT vs. TED) and (i) behavioural and cranial/spinal responses, (ii) brain damage; (iii) fracture classification and (iv) number of shots within different distances from bregma (≤5 mm from bregma; ≥10 mm from bregma). The relationship between cartridge fill and decibel reading of the shot was assessed using linear regression. Analysis was performed in R 3.5.1 [12]. The level of significance for all tests was *p* < 0.05.

## 3. Results

Turkeys had a mean carcase weight of 9.39 ± 2.06 kg (range 5.20–15.60 kg). The carcase weights of birds shot with the TED with no adaptor (12.77 ± 2.02 kg) were significantly heavier than those shot with the hen (8.72 ± 1.41 kg) and modified poult (8.27 ± 1.94 kg) configurations (*p* < 0.05). Carcases of birds shot with the SAT (9.79 ± 1.86 kg) were not significantly different than those of other treatments (hen *p* = 0.43; modified poult *p* = 0.316; no adaptor *p* = 0.10). Both NPCB guns had shot volumes that ranged from 84.1 to 95.4 dB, with the mean for the SAT of 91.1 ± 3.5 dB and 91.4 ± 2.1 dB for the TED, there was no significant difference in dB between the guns (*p* = 0.77). There was no significant relationship between cartridge fill and decibel reading of the SAT shot (r^2^ = 0.03, *p* = 0.40). With continued use of the SAT, a decline in shot volume was apparent (r^2^ = 0.18, *p* = 0.03).

### 3.1. Behaviour and Shot Position

Immediately after stunning all birds showed cessation of rhythmic breathing and loss of neck and beak tension (Table 1). One bird shot with the TED with the Hen configuration showed a positive nictitating membrane reflex in one eye with no other positive reflexes. As a precaution this bird was head-only electrically stunned and bled. Twenty-six percent (*n* = 7) of birds shot with the SAT displayed eyeball rotation, compared to 2% (*n* = 1) with the TED (*p* = 0.01). Tonic and clonic convulsions were observed in all animals, although they were delayed by up to 3 s after the shot in four (9%) of the birds shot with the TED (*n* = 1 hen and *n* = 3 modified poult configurations). None of the birds that had delayed convulsions showed any signs of incomplete concussion. The interval between stunning and exsanguination was ≤ 10 s in all cases.

**Figure 2:**
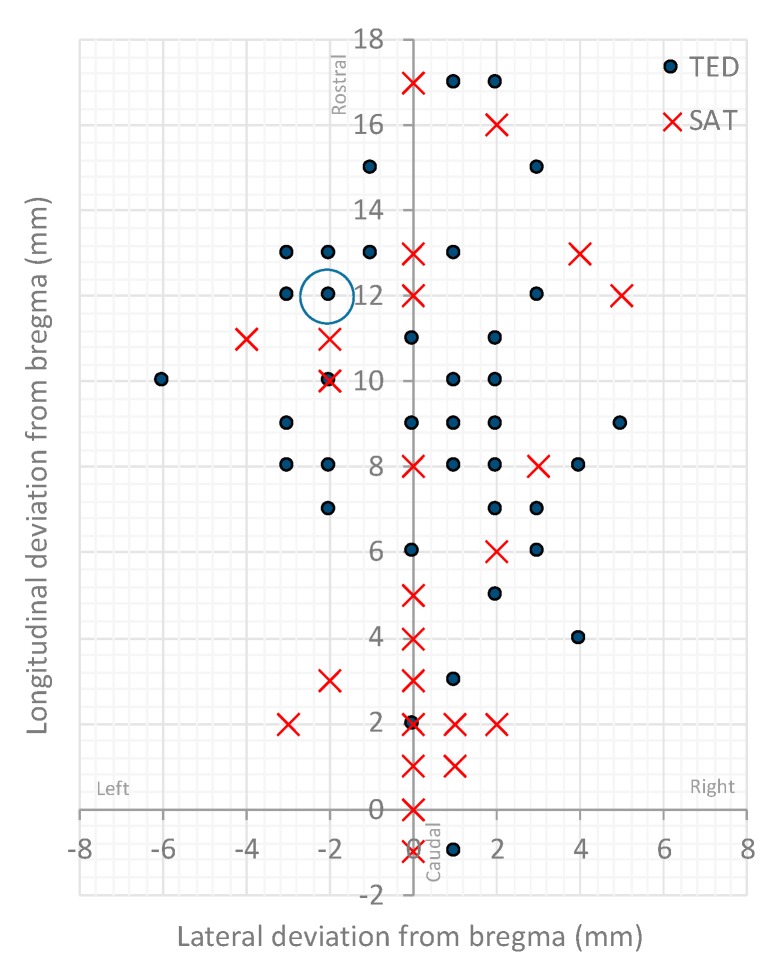
Centre point of bolt impact site relative to bregma (0 mm). Solid circles represent turkeys shot with the Turkey Euthanasia Device (TED) (data for the different TED configurations were pooled), and crosses with the CASH^®^ Small Animal Tool (SAT). The blue (yellow filled) circle indicates the bird which had a positive nictitating membrane reflex. Note some data points are overlapping.

There was substantial variation in shot position between turkeys and captive bolt gun treatments. Data on shot position between the three TED configurations were pooled, as there were no trends in entry site. Shot impact positions ranged from 1 mm caudal to 17 mm rostal of bregma and between 6 and 5 mm left and right of midline respectively (Figure 2). There was a significant difference between treatment groups in the number of shots ≤5 mm from bregma along both axes, with 52% (*n* = 14) and 11% (*n* = 5) shot for the SAT and TED respectively (*p* = 0.01). The animals shot with the TED that showed eyeball rotation, nictitating membrane reflex and delayed convulsions (*n* = 6) after the shot, 67% (*n* = 4) were shot ≥10 mm from bregma.

### 3.2. Pathology

Of the 72 birds stunned with the captive bolts, nine were discounted from the pathology assessment (four macro and nine microscopic). For macroscopic assessment this was due to one bird being restunned with head-only electrical stunning, and three birds damaged during processing of the head. Meanwhile, a further five birds were excluded from histopathological assessment (in total nine) due to processing errors. This included the bird that had a positive nictitating membrane reflex.

#### 3.2.1. Macroscopic Damage

The majority of the birds had skull depression fractures with some radiating from the shot depression. All birds shot with the SAT (*n* = 26/26, one bird discounted from fracture analysis) and 93% (*n* = 40/43, two birds discounted from fracture analysis) of those shot with the TED had depressed fractures of the dorsal surface of the cranium. Seven percent (*n* = 3/43) of birds shot with the TED had no macroscopically visible skull fractures. Eighty-eight percent (*n* = 23/26) of birds shot with the SAT had fractures that were classified as moderate to severe compared to 28% (*n* = 12/43) that were shot with the TED (*p* < 0.01). Of the birds shot with the TED 64% (*n* = 7/11), 33% (*n* = 2/6) and 12% (*n* = 3/26) had moderate to severe skull fractures with the modified poult, none and hen adaptor configurations respectively.

**Figure 3:**
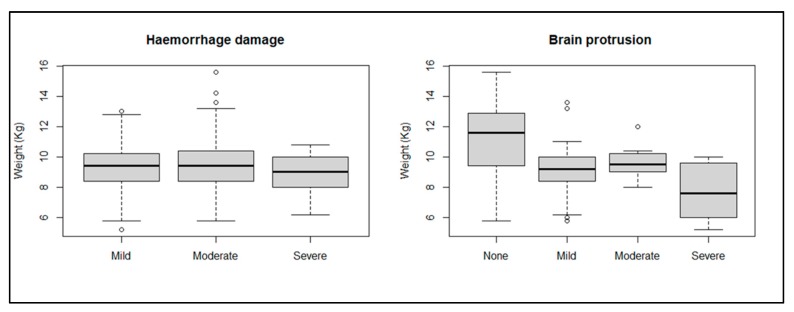
Influence of carcass weight on severity of haemorrhagic damage and brain protrusion.

All birds shot with the different NPCB/configurations had moderate/severe gross damage to the hyperpallium layer over the cerebrums (Table 2). For other structures of the cerebrum the TED with the modified poult adaptor produced the most extensive damage, followed by the SAT. The TED with no adaptor produced the least extensive damage to the inner structures of the cerebrum, though this combination was only used on the larger heads of male birds. Carcass weight was not correlated with haemorrhage damage (*p* = 0.44) but was correlated with brain protrusion (*p* = 0.05), with heavier birds less likely to present brain protrusion (Figure 3).

The SAT and TED with the modified poult configuration produced the most extensive gross damage to the thalamus and hindbrain regions, with 75% and 64% of birds respectively having moderate/severe damage in the thalamus (Table 3). The hindbrain had moderate/severe damage in 52% and 55% of birds shot with the SAT and TED/poult configuration respectively. Male turkeys shot with the TED with no adaptor had the least amount of moderate/severe damage to the thalamus (33%), midbrain (17%), and hindbrain (17%) compared to other treatments. Comparisons between males and females could not be made as male turkeys were only shot with the TED with no adaptor. The bird shot with the TED/hen configuration that had a positive nictitating membrane reflex had mild to severe damage to the structures of the cerebrum, with only mild and moderate damage to the thalamus/midbrain and cerebellum respectively. This bird had no detectable gross damage to the hindbrain structures.

#### 3.2.2. Microscopic Damage

**Figure 4:**
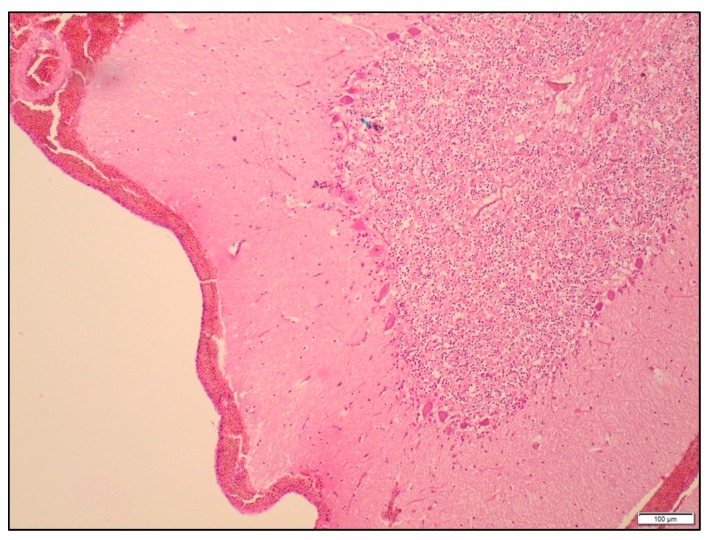
Focal subarachnoid haemorrhage over the cerebellum in a turkey shot with the SAT, ×240 magnification. Haematoxylin and eosin.

**Figure 5:**
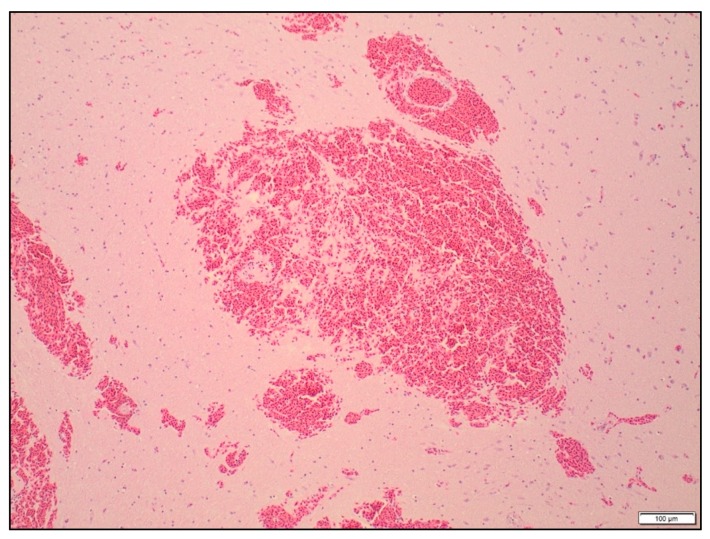
Petechial haemorrhage in the midbrain in a turkey shot with the TED, ×240 magnification. Haematoxylin and eosin.

All brain slices had some degree of subarachnoid haemorrhage over the surface of the brain (Figure 4). There was no significant difference in the severity of the haemorrhage between the treatments (*p* = 0.43). Petechial haemorrhage was evident in the cerebrums, thalamus and midbrain of all the birds, regardless of treatment (Table 4 and Figure 5). The frequency of petechial haemorrhage in the pons and medulla was slightly less in SAT shot birds (76% and 71% respectively) compared to those shot with the different configurations of the TED (ranging from 81 to 100%), however this difference was not significant. In addition to the petechiae, 100% of the brain slices in all groups showed congested blood vessels in all examined regions of the brain.

Microscopic bone fragments were found in the brains of birds shot with the SAT (48%) and the TED (adaptors: hen 38% (10/26), poult 60% (6/10) and none 67% (4/6)). The dorsal surface of the cerebrum was the most common location, however microscopic fragments were also found in the thalamus, midbrain, pons, and medulla.

## 4. Discussion

Annually between 14.3–15.6 million turkeys are slaughtered in the UK [13], with 5.8 million birds being slaughtered during the peak period between October and January [14]. A significant proportion of these birds are slaughtered and processed by small-scale seasonal producers using head-only constant voltage stunning equipment [15]. With a widespread acknowledgment of the limitations of currently used constant voltage head-only electrical stunning systems [1,16] there has been a refocus on the captive bolt as an alternative method for small scale seasonal turkey producers.

This study is the first to compare the pathophysiology of captive bolt injury between the SAT and the TED in commercially slaughtered turkeys. Both guns were effective in inducing unconsciousness. Only one bird shot with the TED/hen configuration had a positive nictitating membrane reflex in one eye after the shot. This bird displayed no other signs of recovery and as a precaution was head-only electrically stunned and bled. The shot position was close to midline but rostral. It was found to have only mild damage to the thalamus and midbrain and no macroscopic damage to the hindbrain structures (pons and medulla). It is unlikely this bird was fully conscious, rather it was showing signs of incomplete concussion. Unfortunately, due to slide processing errors, histopathological assessment of damage could not be performed on this animal.

Variation in NPCB shot position has been previously reported to result in incomplete concussion in turkeys [4]. However, in the current study despite variation in shot entry sites (1 mm caudal to 17 mm rostral of bregma and between 6 and 5 mm left and right of midline), no turkeys showed signs of recovery of consciousness. Caution must be taken with this finding as it does not suggest that correct shot position is not essential. Rather this finding suggests that when shot within these boundaries that unconsciousness can be induced. It is still recommended for effective stunning and animal welfare that birds are shot in the ideal position. This is when the muzzle is positioned perpendicularly on midline, between the eyes and the ears [2,4,5], this position will maximise damage to the underlying brain structures.

Generally, the SAT caused more severe damage to the diencephalon and hindbrain structures compared to the TED adaptors. Only 16% of birds shot with the SAT had no physical trauma to the hindbrain structures compared to 42%, 18%, and 67% of birds shot with the TED with hen, poult, and no adaptor respectively. In addition, there were more extensive skull fractures with the SAT compared to the TED with all three adaptors. Eighty-eight percent (*n* = 23) of birds shot with the SAT had fractures that were classed as moderate to severe compared to 28% (*n* = 12) with the TED. The variation in the severity of damage likely relates to the differences between the guns in mean peak kinetic energy. Gibson et al. [4], reported that the mean peak kinetic energy of the SAT was 75.9 ± 4.5 J compared to 28.4 ± 0.4 J for the TED. However, despite the differences in severity of damage and kinetic energy, both captive bolt gun models and the three variations of the TED, were sufficient to produce unconsciousness in the turkeys.

Studies have reported variations in powerload fill weight affecting peak velocity and delivered kinetic energy. This variation was between and within batches of the cartridges recommended for the SAT [17,18]. Grist et al. [18] reported that out of 41 Accles and Shelvoke 1.00 grain cartridges, four were defective (10%) and only delivered 4–14 J of kinetic energy. In the current study, to test for this variation, discharge noise was recorded as a measure of cartridge performance for each shot. Previously, Gregory et al. [19] reported an association between decibel reading and signs of incomplete concussion in captive bolt stunned young bulls. Meanwhile, Gibson et al. [4] anecdotally reported a ‘soft sounding’ shot as being associated with incomplete concussion (based on EEG analysis) in a turkey shot in the correct position with the SAT. However, in the current study there was no association between shot decibel level, cartridge fill (SAT only) and performance in inducing unconsciousness. Potentially this may relate to the small sample size in the current study, making it unable to detect a rare event. Only 27 birds were shot with the SAT and only cartridges weighing above 0.723 g were selected. If the sample size was increased and lower fill cartridges were included then there would be the potential for stunning failures associated with propellent fill. The focus of the study was not the assessment of cartridge performance, rather the investigation of the different stunning devices and as such cartridges with lower weights were excluded.

In the study, a weak but significant relationship was found between continued use of the SAT and a decline in shot volume. This was unlikely to be due to cartridge fill or heat build-up as the cartridge order was randomised and the SAT was not continually used, being rotated with other treatments. It is more likely that the decrease in shot volume related to the build-up of carbon deposits within the SAT during the study, which may have resulted in the piston end of the bolt not fully retracting into the expansion chamber. This may have caused enlargement of the expansion chamber and a decrease in bolt velocity which has been previously reported for penetrating captive bolt gun models [17]. The incomplete retraction of the SAT bolt has been anecdotally observed by the authors during repeat use on farms and abattoirs. Although in this study there was no order effect in SAT performance, operators should be advised to check that the bolt has fully retracted prior to use on live animals.

In the study there was widespread microscopic petechial haemorrhage in the majority of brain slices, in the absence of macroscopic damage. This highlights the potential limitation of gross macroscopic pathological analysis when assessing injury associated with concussive trauma. However, it was not possible to associate the level of trauma (macro- and microscopic) with outcome as there were no cases of full recovery and only one case of potential incomplete concussion.

A potential limitation of the study was the processing of the pathology samples. In the study the skull and fixed brain were sectioned transversely in-situ together. This approach, while preventing the deformation of the brains during extraction, could have resulted in the introduction of bone fragments into the brain during the sawing process. Potentially this was the source of the microscopic fragments found in the thalamus, midbrain, pons, and medulla during histopathological analysis. Removal of the brains prior to fixation would have prevented this potential biasing factor. However, fixation in-situ has the advantage of reducing the likelihood of displacement of bone fragments and haemorrhage from within the brain during extraction, and also maintains the shape of the brain, preventing gravity induced flattening of brain tissues, which is commonly seen when fixing in formalin pots.

In the study the larger birds dispatched with the TED were males and were generally shot without an adaptor. This made comparisons between gun/adaptor type and bird weight impossible. Ideally, matched groups of birds of different weights and genders would have been stunned with each of the treatments to allow comparison of weight vs. gender vs. treatment type vs. stunning performance. However, this was not possible as the animals were stunned under commercial conditions and it would have been illegal, unacceptable to the producer, and unethical to test configurations of the TED that were likely to produce incomplete concussion. In the UK this type of study would need to be conducted under a Home Office Project License.

For NPCB devices, Annex I of European Council Regulation 1099/2009 on the protection of animals at the time of killing [20] has a special requirement that “*operators shall pay attention to avoid the fracture of the skull*”. In this study, the majority of turkeys assessed for pathology sustained skull fractures to some degree (including hairline fractures in the case of the TED only); only 7% of turkeys shot with the TED and none shot with the SAT had intact skulls with no fractures. The presence of skull fractures did not appear to be to the detriment of stunning efficacy; rather, the SAT and the TED/poult configuration produced the most brain damage, as well as the most severe skull damage.

## 5. Conclusions

In conclusion, the study found that both NPCB guns were effective in inducing unconsciousness in turkeys, regardless of the variations in shot position and the different configurations of the TED. The difference in the severity of brain tissue damage is likely to be related to both the bolt velocity and the respective size of the birds’ heads. The results presented in this paper further support the use of NPCB stunning as an effective on-farm stunning method for both turkey commercial slaughter and on-farm culling, when immediately followed by a secondary killing method.

## Figures and Tables

**Table 1:** Number and percentage of behavioural and cranial/spinal responses after captive bolt shooting with the SAT and TED with the three different configurations.

Configuration	SAT (*n* = 27)	TED		
		Hen (*n* = 28)	Modified Poult (*n* = 11)	None ^†^ (*n* = 6)
Convulsions after the shot	27 (100%)	28 (100%)	11 (100%)	6 (100%)
Normal rhythmic breathing after shot	0 (-)	0 (-)	0 (-)	0 (-)
Positive nictitating membrane reflex	0 (-)	1 (4%) *	0 (-)	0 (-)
Showing nystagmus	0 (-)	0 (-)	0 (-)	0 (-)
Eyeball rotation	7 (26%)	0 (-)	0 (-)	1 (17%)
Presence of neck tension	0 (-)	0 (-)	0 (-)	0 (-)
Presence of beak tension	0 (-)	0 (-)	0 (-)	0 (-)

^†^ All large male birds. * Bird re-stunned with head-only electrical stunning.

**Table 2:** Macroscopic damage in cerebrum structures following non-penetrating captive bolt with SAT (*n* = 25) and TED (*n* = 43).

Regions and Severity	SAT (*n* = 25) *	TED		
		Hen (*n* = 26)	Modified Poult (*n* = 11)	None (*n* = 6) ^†^
Hyperpallium				
None	0 (-)	0 (-)	0 (-)	0 (-)
Mild	0 (-)	0 (-)	0 (-)	0 (-)
Moderate/Severe	25 (100%)	26 (100%)	11 (100%)	6 (100%)
Mesopallium				
None	1 (4%)	0 (-)	0 (-)	0 (-)
Mild	3 (12%)	3 (12%)	0 (-)	2 (33%)
Moderate/Severe	21 (84%)	23 (89%)	11 (100%)	4 (67%)
Nidopallium				
None	2 (8%)	0 (-)	1 (9%)	0 (-)
Mild	4 (16%)	9 (35%)	0 (-)	3 (50%)
Moderate/Severe	19 (76%)	17 (65%)	10 (90%)	3 (50%)
Stratum				
None	1 (4%)	1 (4%)	0 (-)	1 (17%)
Mild	6 (25%)	9 (35%)	1 (9%)	3 (50%)
Moderate/Severe	17 (71%)	16 (62%)	10 (91%)	2 (33%)
Pallidum				
None	3 (13%)	7 (27%)	2 (18%)	2 (33%)
Mild	6 (25%)	6 (23%)	2 (18%)	2 (33%)
Moderate/Severe	15 (63%)	13 (50%)	7 (66%)	2 (33%)

^†^ All large male birds. * In one bird (SAT) it could not be determined if there was damage to the stratum or pallidum.

**Table 3:** Macroscopic damage in thalamic and hindbrain structures following non-penetrating captive bolt with SAT (*n* = 25) and TED (*n* = 43).

Regions and Severity	SAT (*n* = 25) *	TED		
		Hen (*n* = 26)	Modified Poult (*n* = 11)	None (*n* = 6) ^†^
Thalamus				
None	3 (13%)	6 (23%)	0 (-)	2 (33%)
Mild	3 (13%)	7 (27%)	4 (36%)	2 (33%)
Moderate/Severe	18 (75%)	13 (50%)	7 (64%)	2 (33%)
Midbrain				
None	8 (33%)	6 (23%)	3 (27%)	2 (33%)
Mild	9 (38%)	12 (46%)	7 (64%)	3 (50%)
Moderate/Severe	7 (29%)	8 (31%)	1 (9%)	1 (17%)
Hindbrain				
None	4 (16%)	11 (42%)	2 (18%)	4 (67%)
Mild	8 (32%)	8 (31%)	3 (27%)	1 (17%)
Moderate/Severe	13 (52%)	7 (27%)	6 (55%)	1 (17%)
Cerebellum				
None	1 (4%)	6 (23%)	3 (27%)	1 (17%)
Mild	14 (56%)	10 (39%)	5 (46%)	3 (50%)
Moderate/Severe	10 (40%)	10 (39%)	3 (27%)	2 (33%)

^†^ All large male birds. * In one bird (SAT) it could not be determined if there was damage to the thalamus or midbrain.

**Table 4:** Presence of microscopic petechial haemorrhage across 6 areas of the brain, with the SAT and TED. Nine birds were excluded due to electrical stunning/brain removal (*n* = 4) or due to slide processing errors (*n* = 5).

Regions	SAT (*n* = 21)	TED		
		Hen (*n* = 26)	Modified Poult (*n* = 10)	None (*n* = 6) ^†^
Cerebrum	21 (100%)	26 (100%)	10 (100%)	6 (100%)
Thalamus	21 (100%)	26 (100%)	10 (100%)	6 (100%)
Midbrain	21 (100%)	26 (100%)	10 (100%)	6 (100%)
Pons	16 (76%)	23 (88%)	9 (90%)	6 (100%)
Medulla	15 (71%)	21 (81%)	9 (90%)	5 (83%)
Cerebellum	20 (95%)	26 (100%)	9 (90%)	6 (100%)

^†^ All large male birds.

## References

[1] Gibson T.J., Taylor A.H., Gregory N.G. (2016). Assessment of the effectiveness of head only and back-of-the-head electrical stunning of chickens. Br. Poult. Sci..

[2] Erasmus M.A., Lawlis P., Duncan I.J., Widowski T.M. (2010). Using time to insensibility and estimated time of death to evaluate a nonpenetrating captive bolt, cervical dislocation, and blunt trauma for on-farm killing of turkeys. Poult. Sci..

[3] Raj A.B.M., O’Callaghan M. (2001). Evaluation of a pneumatically operated captive bolt for stunning/killing broiler chickens. Br. Poult. Sci..

[4] Gibson T.J., Rebelo C.B., Gowers T.A., Chancellor N.M. (2018). Electroencephalographic assessment of concussive non-penetrative captive bolt stunning of turkeys. Br. Poult. Sci..

[5] Woolcott C., Torrey S., Turner P., Serpa L., Schwean-Lardner K., Widowski T. (2018). Evaluation of Two Models of Non-Penetrating Captive Bolt Devices for On-Farm Euthanasia of Turkeys. Animals.

[6] Erasmus M.A., Turner P.V., Nykamp S.G., Widowski T.M. (2010). Brain and skull lesions resulting from use of percussive bolt, cervical dislocation by stretching, cervical dislocation by crushing and blunt trauma in turkeys. Vet. Rec..

[7] Reiner A., Perkel D.J., Bruce L.L., Butler A.B., Csillag A., Kuenzel W., Medina L., Paxinos G., Shimizu T., Striedter G. (2004). Revised nomenclature for avian telencephalon and some related brainstem nuclei. J. Comp. Neurol..

[8] Jarvis E.D., Güntürkün O., Bruce L., Csillag A., Karten H., Kuenzel W., Medina L., Paxinos G., Perkel D.J., Shimizu T. (2005). Avian brains and a new understanding of vertebrate brain evolution. Nat. Rev. Neurosci..

[9] Gibson T.J., Whitehead C., Taylor R., Sykes O., Chancellor N.M., Limon G. (2015). Pathophysiology of penetrating captive bolt stunning in Alpacas (Vicugna pacos). Meat Sci..

[10] Gibson T.J., Bedford E.M., Chancellor N.M., Limon G. (2015). Pathophysiology of free-bullet slaughter of horses and ponies. Meat Sci..

[11] Gibson T.J., Ridler A.L., Lamb C.R., Williams A., Giles S., Gregory N.G. (2012). Preliminary evaluation of the effectiveness of captive-bolt guns as a killing method without exsanguination for horned and unhorned sheep. Anim. Welf..

[12] Team R.d.c. (2008). A Language and Environment for Statistical Computing.

[13] FAOSTAT Turkey Meat Production. http://www.fao.org/faostat/en/#data/QL.

[14] DEFRA Poultry and Poultry Meat Statistics. https://www.gov.uk/government/collections/poultry-and-poultry-meat-statistics.

[15] Rebelo C. (2019). Personal communication.

[16] HSA (2012). Through the years: HSA training courses for smallholders who slaughter poultry on farm. Scientific Workshop on Head-only Electrical Stunning of Turkeys. Caring Beyond the Farm Gate: Humane Slaughter Association Annual Report 2011–2012.

[17] Gibson T.J., Mason C.W., Spence J.Y., Barker H., Gregory N.G. (2015). Factors affecting penetrating captive bolt gun performance. J. Appl. Anim. Welf. Sci..

[18] Grist A., Lines J.A., Bock R., Knowles T.G., Wotton S.B. (2019). An Examination of the Performance of Blank Cartridges Used in Captive Bolt Devices for the Pre-Slaughter Stunning and Euthanasia of Animals. Animals.

[19] Gregory N.G., Lee C.J., Widdicombe J.P. (2007). Depth of concussion in cattle shot by penetrating captive bolt. Meat Sci..

[20] EU (2009). Council Regulation (EC) No 1099/2009 of 24 September 2009 on the protection of animals at the time of killing. Off. J. Eur. Union.

